# Rosai–Dorfman disease manifesting as epibulbar and orbital tumor: A case report and literature review

**DOI:** 10.1097/MD.0000000000018757

**Published:** 2020-01-10

**Authors:** Qing Huang, Hong Cai, Weimin He

**Affiliations:** Department of Ophthalmology, West China Hospital of Sichuan University, Chengdu, Sichuan, China.

**Keywords:** epibulbar tumor, orbital tumor, Rosai–Dorfman disease

## Abstract

**Rationale:**

Rosai-Dorfman disease (RDD) is a rare nonmalignant cell histiocytosis. Here, we report a rare, unusual clinical presentation of epibulbar and orbital tumor as a manifestation of RDD. We also review the literatures on clinical cases of orbital RDD.

**Patient concerns:**

A 44-year-old Chinese male was admitted with a 3-month history of eye redness, subconjunctival mass, and diplopia.

**Diagnosis:**

An initial diagnosis of epibulbar and orbital tumor was made according to the clinical symptoms, signs, and pre-operative contrast-enhanced computerized tomography results.

**Interventions:**

The mass was completely resected and pathology confirmed the RDD diagnosis. The patient received steroids after surgery.

**Outcomes:**

The patient recovered well on 18-month follow-up.

**Conclusion:**

This appears to be the first report of RDD manifesting as epibulbar and orbital tumor. Pre-operative diagnosis of RDD remains challenging. When the lesion causes diplopia, surgical resection is the most effective treatment.

## 1 Introduction

Rosai–Dorfman disease (RDD) is a rare, idiopathic, non-neoplastic histioproliferative disease of unknown etiology. Extranodal RDD occurs infrequently but can be the predominant or only manifestation; the most common extranodal sites are the eyes, ocular adnexa, and orbit.^[1]^ Ocular RDD may simulate malignancy but is typically benign and self-limited.^[2]^ In this work we report for the first time a case of extranodal RDD manifesting as epibulbar and orbital tumor with no systemic involvement.

## 2 Case presentation

**Figure 1:**
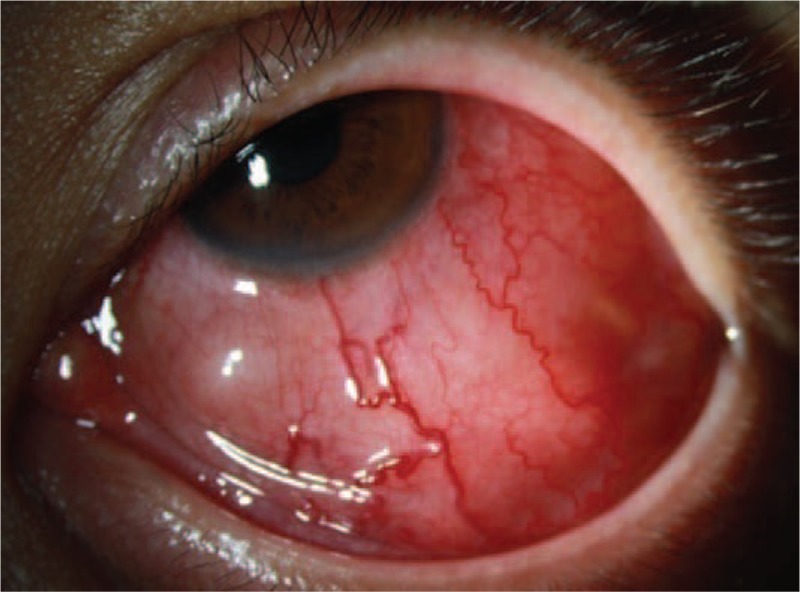
Slit-lamp image. Inferior subconjunctival mass with conjunctival hyperemia.

**Figure 2:**
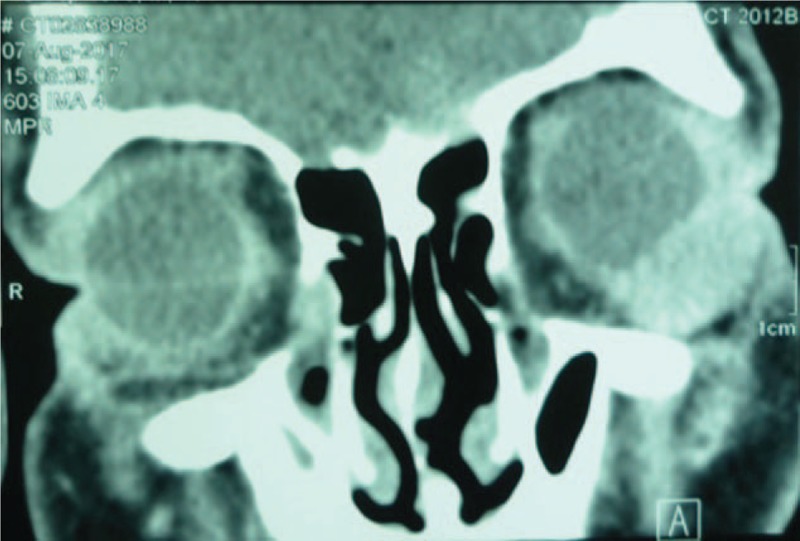
Coronal slice obtained by contrast-enhanced computed tomography. A soft tissue mass was observed in the inferotemporal side of the orbit, and the nearby eyeball was pressed.

A 44-year-old male presented with left eye redness and a subconjunctival mass, complicated with diplopia, this had persisted for the previous 3 months. The patient had provided informed consent for publication of the case. Visual acuity was 20/25 in the right eye and 20/100 in the left eye. Slit lamp microscopy showed conjunctival hyperemia in the left eye with a slight elevation, suggesting a subconjunctival mass (Fig. 1). Intraocular inflammatory reaction was not found. Intraocular pressure was 10 mmHg in both eyes. The retina was normal by ophthalmoscopy examination. Contrast-enhanced computerized tomography (CT) of the eye showed a soft tissue mass in the inferotemporal side of the orbit with a maximal section of 25 × 25 mm, which was pressing the peripheral eyeball (Fig. 2). Investigation of systemic parameters was unremarkable, and all blood tests were within the normal ranges.

**Figure 3:**
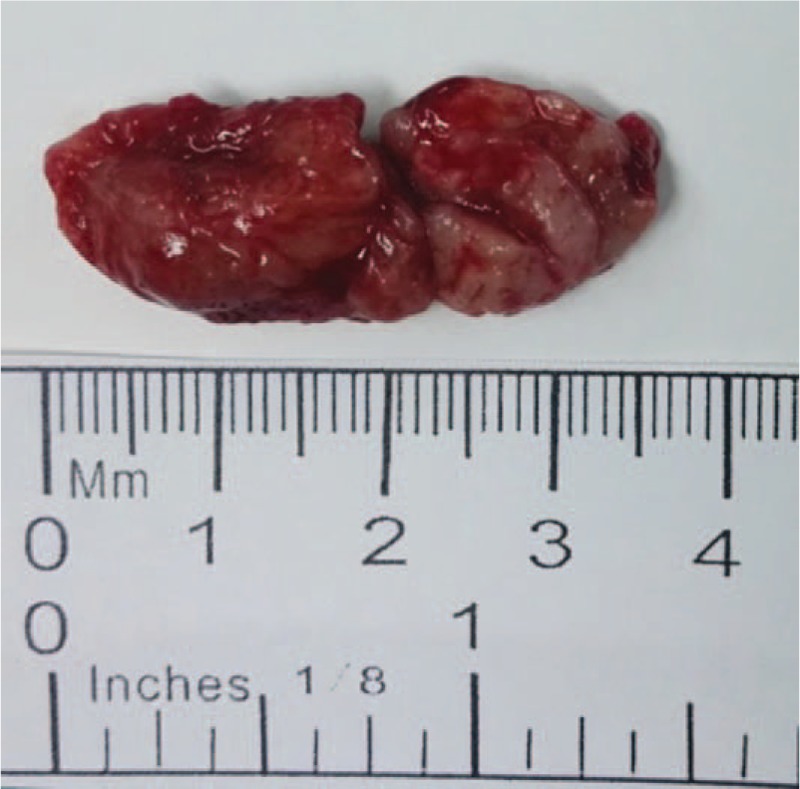
Image of the excised mass. The cut section of the specimen appeared solid and gray.

**Figure 4:**
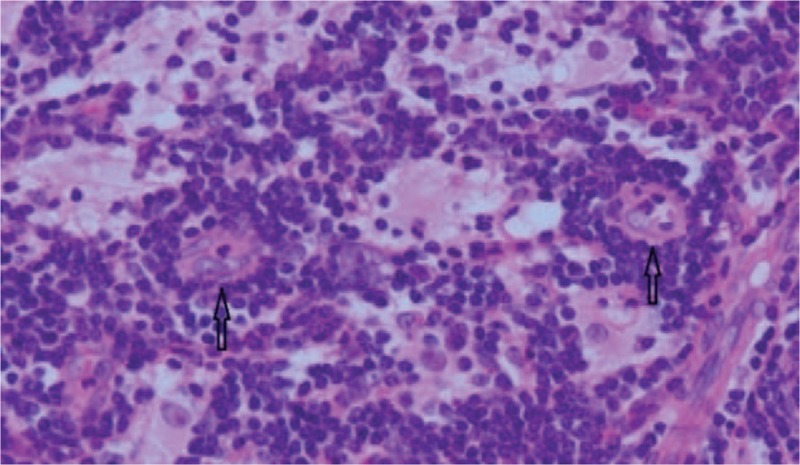
Image of a histopathologic section stained with hematoxylin and eosin (×200). Plasma cells and neutrophil infiltration were observed. The arrows show areas of emperipolesis, which contain large histocytes with vesicular nuclei and abundant cytoplasm with engulfed lymphocytes and plasma cells.

**Figure 5:**
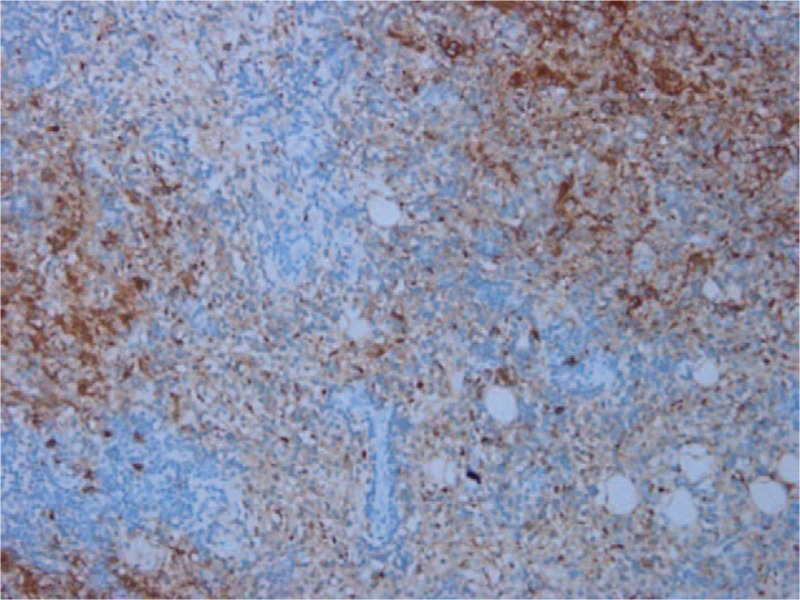
Image of histopathologic section stained with S-100 (×200).

The patient underwent resection surgery. During surgery, we saw that the subconjunctival mass in the inferotemporal corneal limbus extended backwards towards the orbit. The excised mass appeared solid and gray (Fig. 3). Pathological examination showed fibrous and lymphoid tissue hyperplasia and lymphatic follicle formation. Areas of emperipolesis were noted, in which sparse plasma cells and neutrophil infiltration were observed (Fig. 4). The biopsy stained positive for S-100 (Fig. 5) and immunoglobulin G4 (IgG4) (focal area about 50/HPF). Gene rearrangement assays using Polymerase Chain Reaction and Gene Scan did not show a cloning amplification peak for IgH and IgK genes. The pathologic features and immunostaining results were consistent with extranodal RDD. We were also careful to exclude IgG4 disease, since both diseases show mixed inflammation that typically includes reactive follicles and abundance of plasma cells, and can be associated with elevated numbers of IgG4-positive plasma cells. In our case, the emperipolesis and S-100-positive macrophages supported the diagnosis of RDD instead of IgG4-related disease.^[3]^ The patient underwent a thorough systemic evaluation and the results were unremarkable.

**Figure 6:**
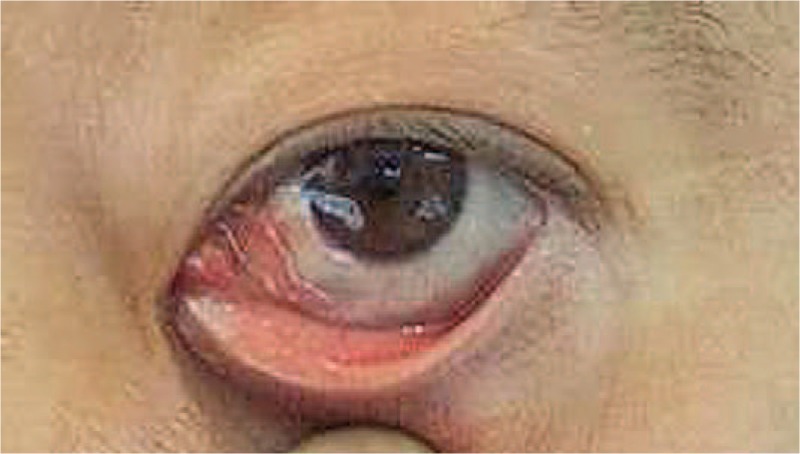
Image of external eye appearance at 18 months after surgery.

The patient was prescribed prednisolone (30 mg/day). At 18-month follow-up, the patient showed no signs of recurrence, and the diplopia had resolved (Fig. 6).

## 3 Discussion

A search in PubMed was performed to identify previous case reports of orbital RDD. The search terms included “orbit / orbital” and “Rosai–Dorfman disease / sinus histiocytosis with massive lymphadenopathy”. All bibliographic references from the main reports and relevant reviews were screened manually for additional eligible studies. The results were limited to full-text articles published in English. Extracted data included orbital location, patient characteristics (country / nationality, gender and age at onset), other affected sites, treatment, and follow-up.

**Figure 7:**
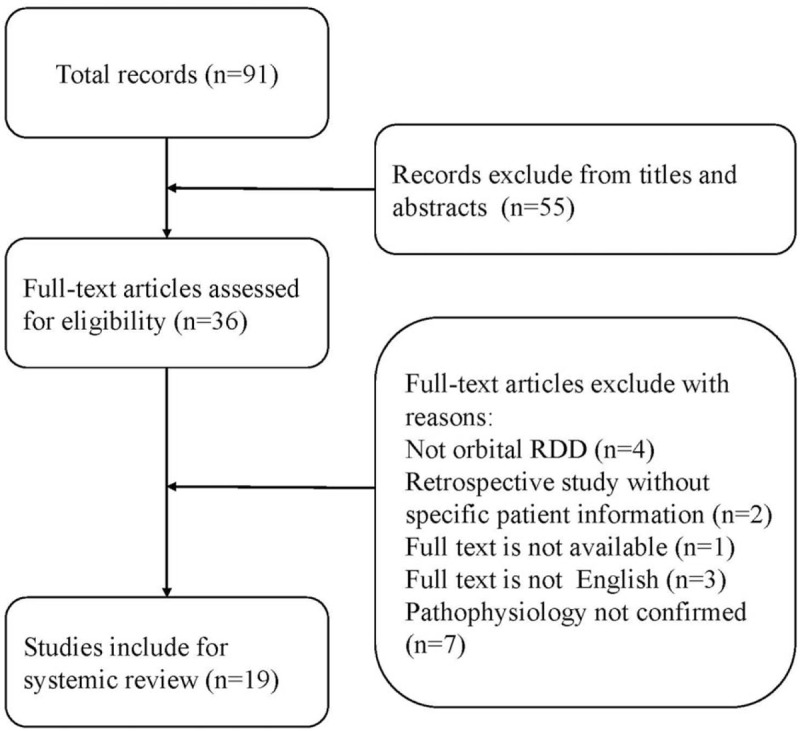
Flowchart of study selection for the literature review.

A total of 91 publications were identified, of which 19^[4–22]^ were available and were finally included in the review, 55 records were excluded from titles and abstracts and 17 were excluded with other reasons (Fig. 7). The total number of patients analyzed was 35 (18 Caucasian, 5 Afro-American, 2 Asian, 10 of unknown ethnicity). The disorder seemed to occur evenly in both genders (19 male and 16 female), and the mean age was 30.8 ± 22.9 years (range 2–78 years). In 18 of 31 patients (58.06%), orbital RDD was the only manifestation, while in 19 of 29 patients (65.52%), manifestations occurred at other sites such as brain, lymph nodes, lung, and skin lesions. Treatment included excision, steroids, radiotherapy, chemotherapy, or combined therapy. After a mean follow-up of 18.4 ± 2.9 months, the condition of 23 of 28 patients (82.14%) resolved or improved after treatment, 4 of 28 patients (14.29%) suffered recurrence, and one patient died due to infection with human immunodeficiency virus (Table 1).

**Table 1:**
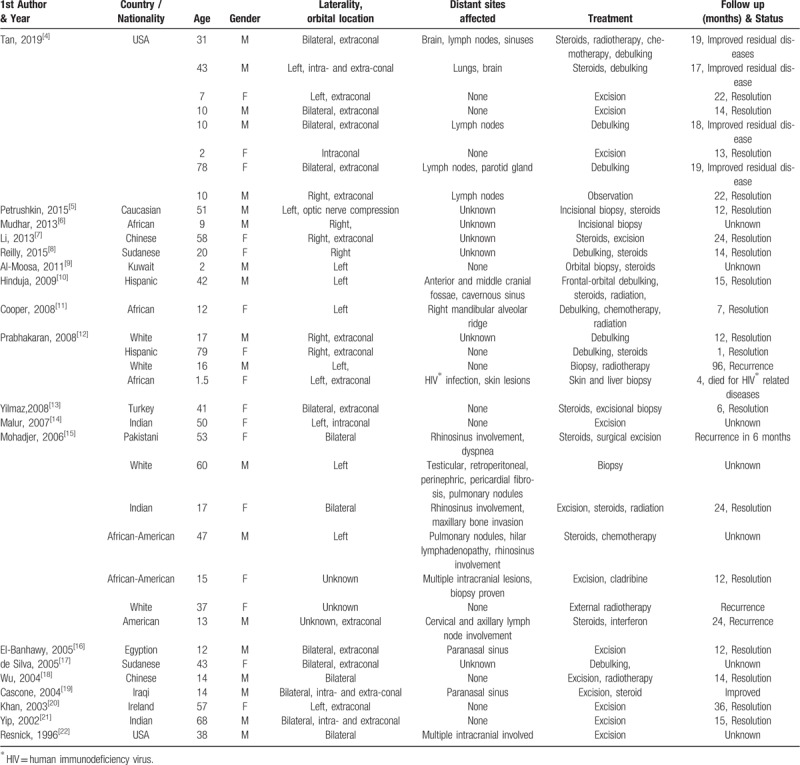
Literature review of case reports of orbital Rosai–Dorfman Disease.

RDD is a rare pseudolymphomatous disorder initially described as a separate entity in 1969 by Rosai and Dorfman using the term sinus histiocytosis with massive lymphadenopathy. RDD is characterized by persistent massive lymphadenopathy and increased numbers of macrophages within lymph node sinuses.^[23,24]^ Extranodal involvement occurs in 43% of cases, with ophthalmic disease observed in 11.5% of patients.^[1]^ The reported ophthalmic manifestations of RDD include orbital, eyelid and epibulbar masses, compressive optic neuropathy, uveitis, scleritis, serous retinal detachments, corneal lesions, and lacrimal sac and / or duct obstruction.^[25]^ Orbital involvement is the most common ophthalmic manifestation, with a prevalence of 2.3%.^[25–26]^

Radiography is critical in the surgical planning of orbital RDD, as well as the investigation of additional potential systemic sites of involvement. Several authors have recommended imaging the neck, chest, and abdomen when there is clinical suspicion.^[25]^ Generally, RDD appears as a homogeneous mass on CT that may be better detailed with magnetic resonance imaging.^[25]^ Bone destruction is rare and has been described in only a few cases.^[12,25,27]^ Histological findings include typical features, such as diffuse lymphoplasmatic infiltration, Russel bodies, foamy histiocytes, and histiocytes with phagocytosed lymphocytes within the cytoplasm (emperipolesis). Immunohistochemistry was positive for S-100, alpha-antichymotrypsin, and the antigens CD1a and CD68.^[23]^ The signs and symptoms of orbital RDD include exophthalmos (most frequent), lagophthalmos, blurred vision, diplopia, conjunctival congestion, dry eye, uveitis, and ocular irritation.^[1]^

The clinical course of RDD is unpredictable. Spontaneous remissions, distant relapses, and involvement of other extranodal sites may occur. Previous work examined 9 extranodal RDD cases and concluded that a lack of lymphadenopathy is characteristic of RDD manifesting as epibulbar tumor, which is consistent with our findings.^[2]^ Surgical excision or debulking, chemotherapy, radiotherapy, and immunosuppressive therapy have all been described in the literatures.^[28]^ When orbital involvement causes symptoms or disfigurement predominantly through the mass effect, the most effective initial treatment may be excision or debulking.^[29]^ Diffuse, residual, or recurrent lesions can be treated with systemic corticosteroids.^[26]^ In the reviewed publications, the prognosis of orbital RDD was good, with 82.14% of patients showing resolution or improvement after treatment.

## 4 Conclusion

We describe what appears to be the first case of extranodal RDD manifesting as epibulbar and orbital tumor. This Chinese patient showed emperipolesis and S-100-positive macrophages, allowing us to exclude IgG4-related disease. Complete surgical excision was performed without complications and with a favorable outcome. Long-term follow-up is required to monitor patients with orbital RDD to prevent vision-threatening complications and recurrence.

## Author contributions

**Data curation:** Qing Huang, Hong Cai.

**Formal analysis:** Qing Huang.

**Validation:** Weimin He.

**Visualization:** Weimin He.

**Writing - Original Draft:** Qing Huang.

**Writing - Review & Editing:** Weimin He.

Qing Huang orcid: 0000-0001-9411-1796.
